# Supplementary material for: Gene regulatory programs underlying diversification of facial ligaments and tendons in zebrafish
Source: Development. 2026 Feb 5;153(2):dev205045. doi: 10.1242/dev.205045 (PMC12912267; doi:10.1242/dev.205045)
Supplement: Supplementary information [file develop-153-205045-s1.pdf]

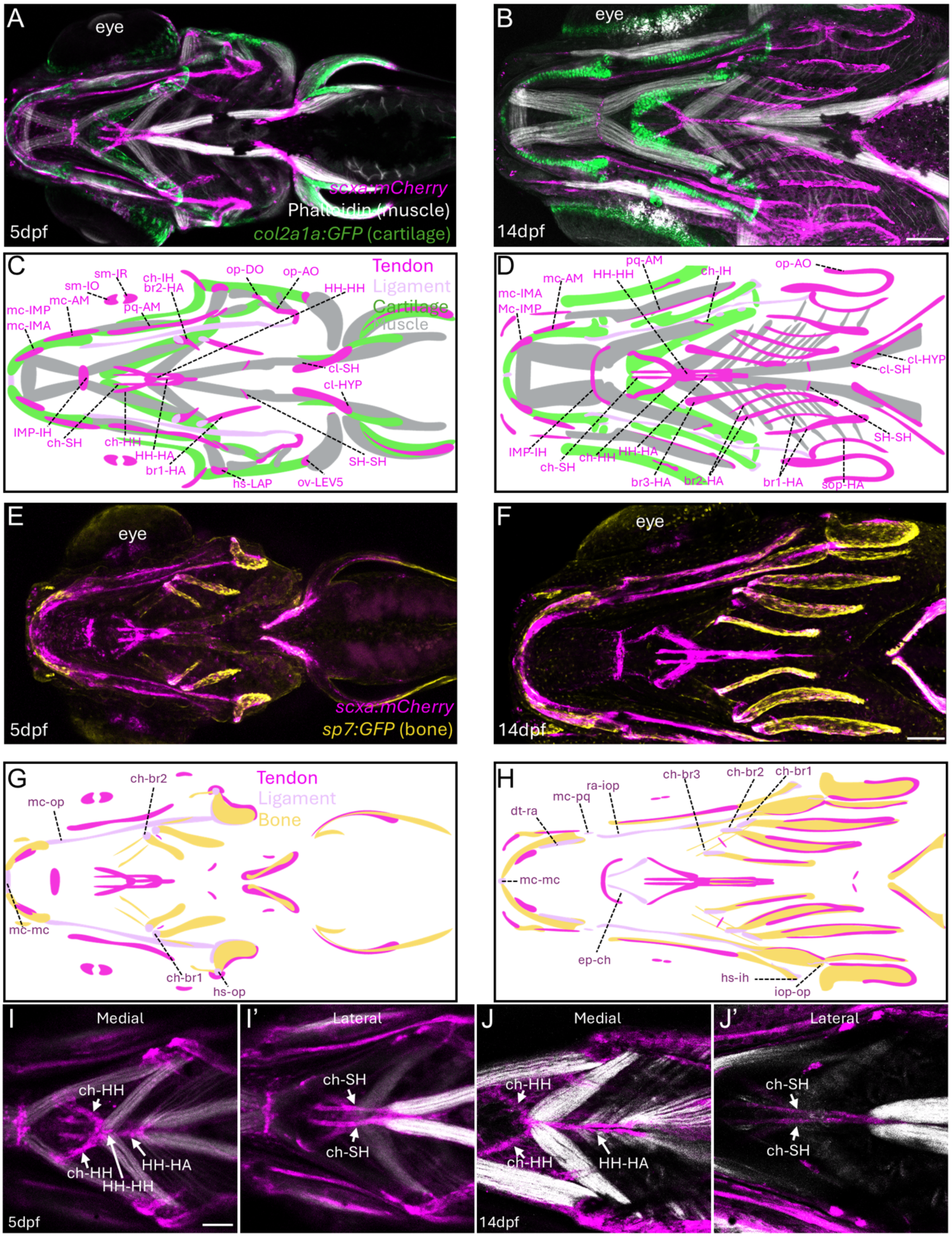

A
eye
scxa:mCherry
Phalloidin (muscle)
col2a1a:GFP (cartilage)
5dpf
B
eye
14dpf
C
Tendon
Ligament
Cartilage
Muscle
sm-IR
sm-IO
ch-IH
br2-HA
op-DO
op-AO
HH-HH
mc-IMP
mc-AM
pq-AM
mc-IMA
cl-SH
cl-HYP
IMP-IH
ch-SH
ch-HH
HH-HA
br1-HA
hs-LAP
ov-LEV5
SH-SH
D
pq-AM
mc-AM
HH-HH
ch-IH
op-AO
mc-IMA
Mc-IMP
cl-HYP
cl-SH
SH-SH
IMP-IH
ch-SH
ch-HH
HH-HA
br3-HA
br2-HA
br1-HA
sop-HA
E
eye
5dpf
scxa:mCherry
sp7:GFP (bone)
F
eye
14dpf
G
Tendon
Ligament
Bone
ch-br2
mc-op
mc-mc
ch-br1
hs-op
H
ra-iop
ch-br3
ch-br2
ch-br1
mc-pq
dt-ra
mc-mc
ep-ch
hs-ih
iop-op
I
Medial
ch-HH
ch-HH
HH-HH
HH-HA
5dpf
I'
Lateral
ch-SH
ch-SH
J
Medial
ch-HH
ch-HH
HH-HA
14dpf
J'
Lateral
ch-SH
ch-SH

Fig. S1. Ventral views of zebrafish facial tendons and ligaments

(A,B) Confocal projections in ventral view show tendons and ligaments (*scxa:mCherry*), muscles (Phalloidin), and cartilages (*col2a1a:GFP*) in the larval zebrafish face at 5 dpf (n=3) and 14 dpf (n=3).

(C,D) Schematics with tendon abbreviations in magenta.

(E,F) Confocal projections in ventral view show tendons and ligaments (*scxa:mCherry*) and bones (*sp7:GFP*) in the larval zebrafish face at 5 dpf (n=3) and 14 dpf (n=3).

(G,H) Schematics with ligament abbreviations.

(I-J) Confocal slices in medial and lateral positions show distinct midline tendons. Scale bars = 100 µm. See Figure 2 for complete list of names and abbreviations.

Development • Supplementary information

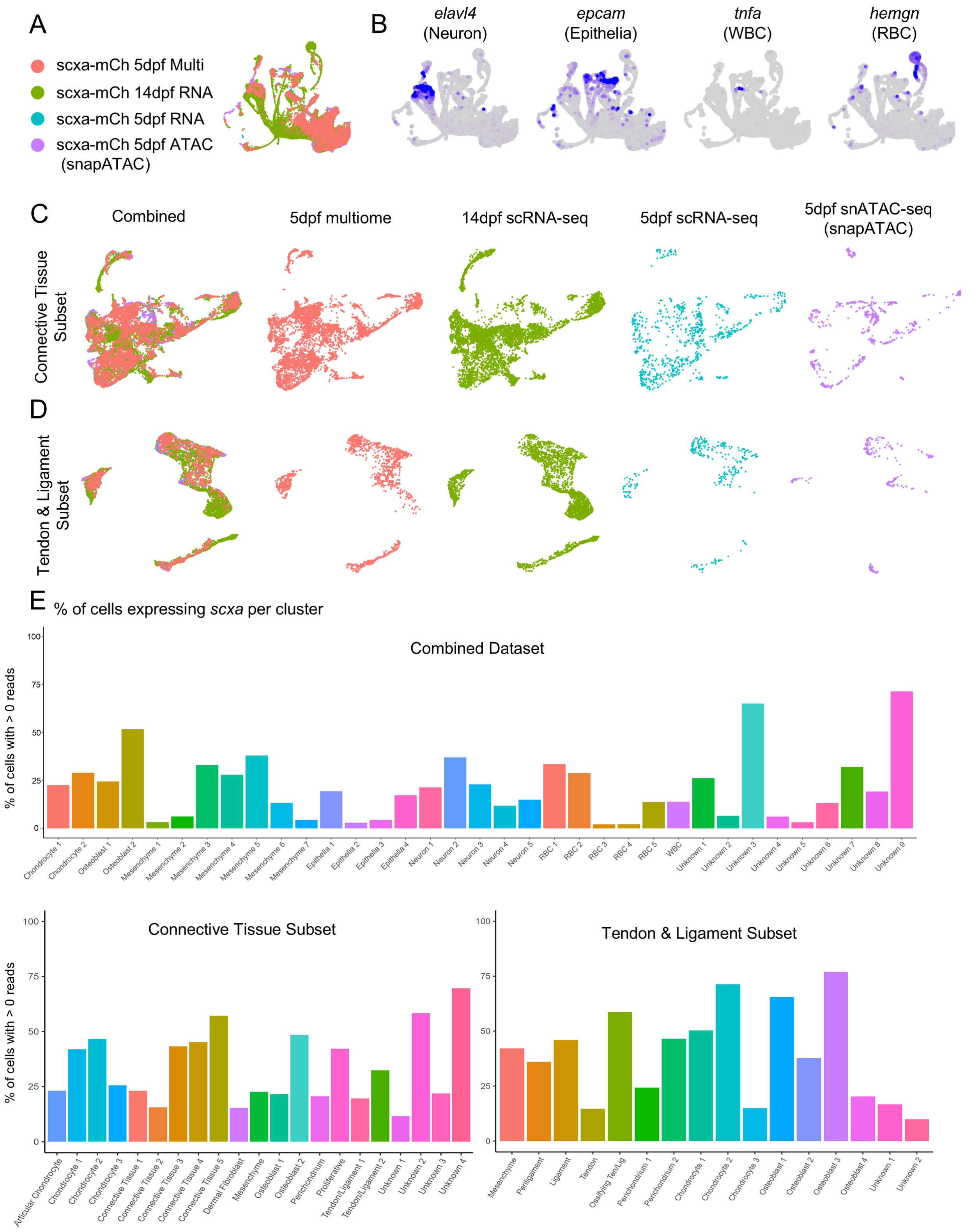

Fig. S2. Additional analysis of single-cell datasets

(A) UMAP of merged libraries from Fig. 3A.

(B) Feature plots from merged libraries show marker genes for neuron (*elavl4*), epithelia (*epcam*), white blood cell (WBC, *tnfa*), and red blood cell (RBC, *hemgn*) clusters.

Development • Supplementary information

(C) UMAPs show merged and individual libraries for the connective tissue and tendon & ligament subsets of Fig. 4A.

(D) UMAPs showing merged and individual libraries for the tendon and ligament subset of Fig. 4A.

(E) Percentage of cells with *scxa* expression per cluster in indicated libraries.

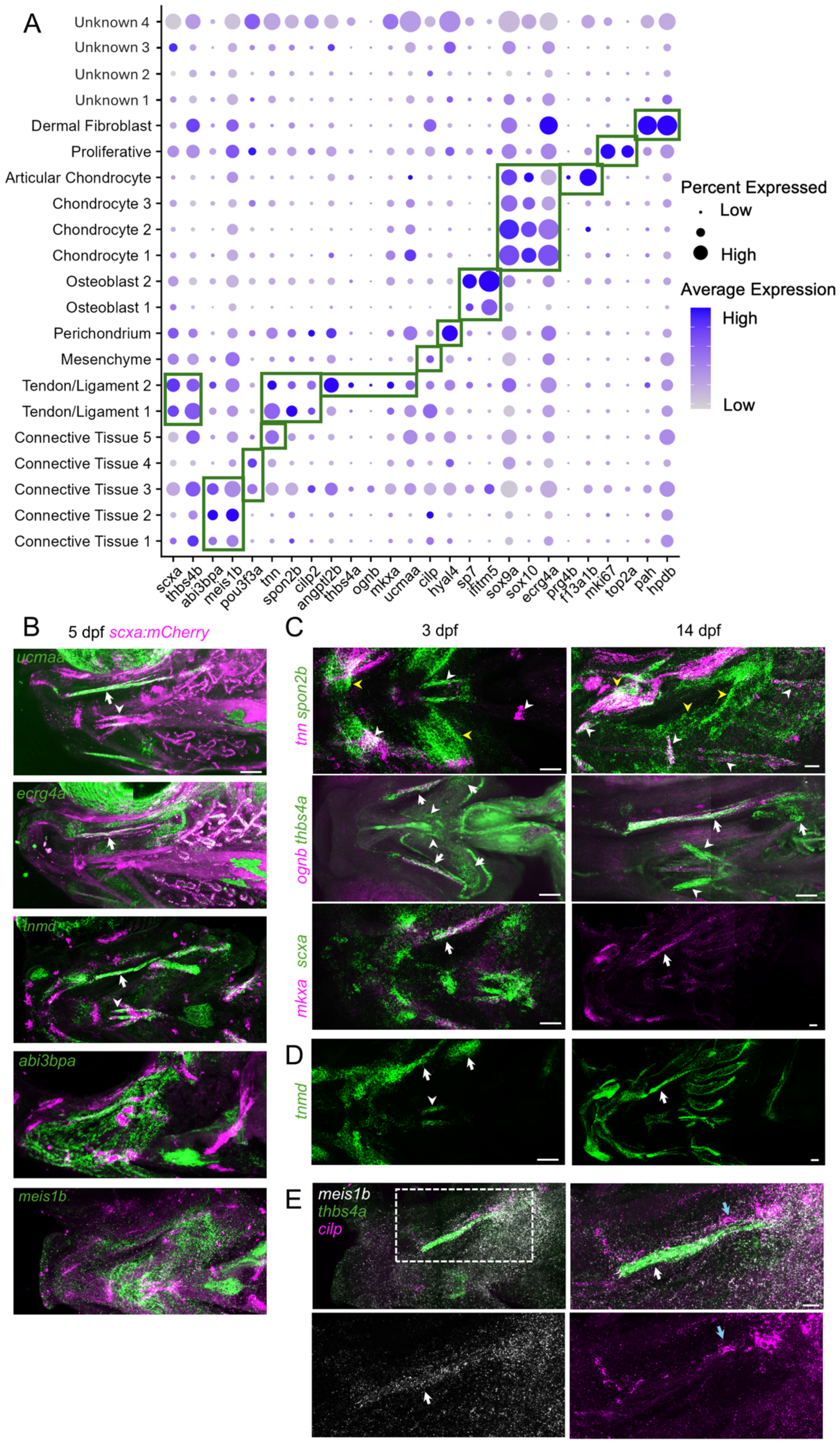
A
Unknown 4
Unknown 3
Unknown 2
Unknown 1
Dermal Fibroblast
Proliferative
Articular Chondrocyte
Chondrocyte 3
Chondrocyte 2
Chondrocyte 1
Osteoblast 2
Osteoblast 1
Perichondrium
Mesenchyme
Tendon/Ligament 2
Tendon/Ligament 1
Connective Tissue 5
Connective Tissue 4
Connective Tissue 3
Connective Tissue 2
Connective Tissue 1
scxa
thbs4b
abi3bpa
meis1b
pou3f3a
tnn
spon2b
cilp2
angptl2b
thbs4a
ognb
mkxa
ucmaa
cilp
hyal4
sp7
ifitm5
sox9a
sox10
ecrg4a
prg4b
f13a1b
mki67
top2a
pah
hpdb
Percent Expressed
Low
High
Average Expression
High
Low
B
5 dpf scxa:mCherry
ucmaa
ecrg4a
tnmd
abi3bpa
meis1b
C
3 dpf
14 dpf
tnn spon2b
ognb thbs4a
mkxa scxa
D
tnmd
E
meis1b
thbs4a
cilp

Fig. S3. Additional marker gene expression analysis

(A) Dot plot of genes used for cluster identification of the connective tissue subset of Fig. 3D. Boxes indicate key markers of each cluster.

(B) RNAscope in situ hybridization, except for HCR in situ hybridization for *ucmaa* and *ecrg4a*, for indicated genes (green) with anti-mCherry staining of *scxa:mCherry* labeling tendons and ligaments. *ucmaa*, n=4; *ecrg4a*, n=3; *tnmd*, n=3; *abi3ba*, n=3; *meis1b*, n=3. **(C,D)** RNAscope in situ hybridization shows expression of indicated genes at 3 and 14 dpf. *tnn/spon2b*, 3 dpf n=3, 14 dpf n=3; *ognb*/*thbs4a*, 3 dpf n=6, 14 dpf n=3; *mkxa/scxa*, 3dpf n=3, 14 dpf n=4; *tnmd*, 3 dpf n=3, 14 dpf n=4.

(E) Triple RNAscope in situ hybridization shows expression of *meis1b*, *thbs4a*, and *cilp* at 5 dpf. Magnified box region centered on the mc-op ligament is shown for merged and individual channels (n=4). Confocal projections are shown in ventral view at 3 dpf and ventrolateral view at 5 and 14 dpf with expression noted for ligaments (white arrows), tendons (white arrowheads), periligament (blue arrows), and perichondrium (yellow arrowheads). Scale bars = 50 μm (B-D) , 20 μm (E).

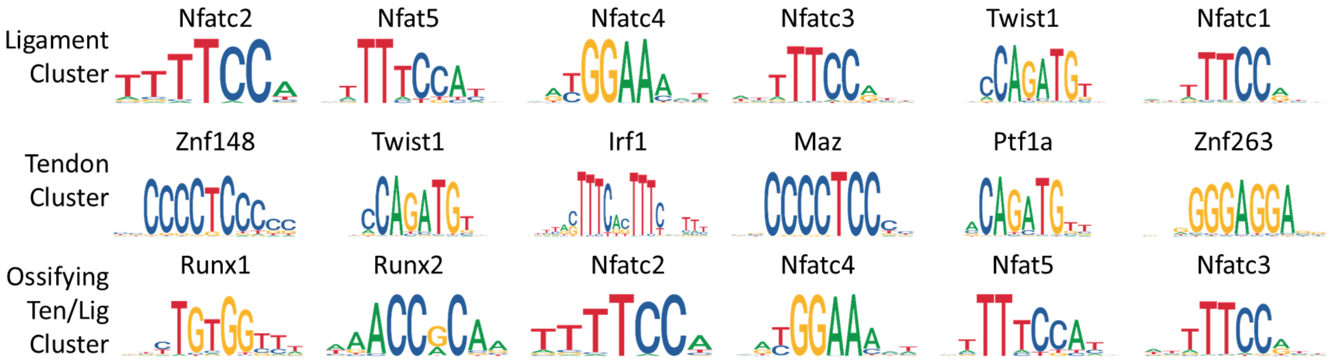

Fig. S4. Motif enrichment analysis

Motif enrichment analysis using Signac highlights the top 6 enriched motifs in the tendon, ligament, and ossifying tendon and ligament clusters, when compared to a randomly selected background of 40,000 motifs from the tendon and ligament subset of Fig. 4A.

Development • Supplementary information

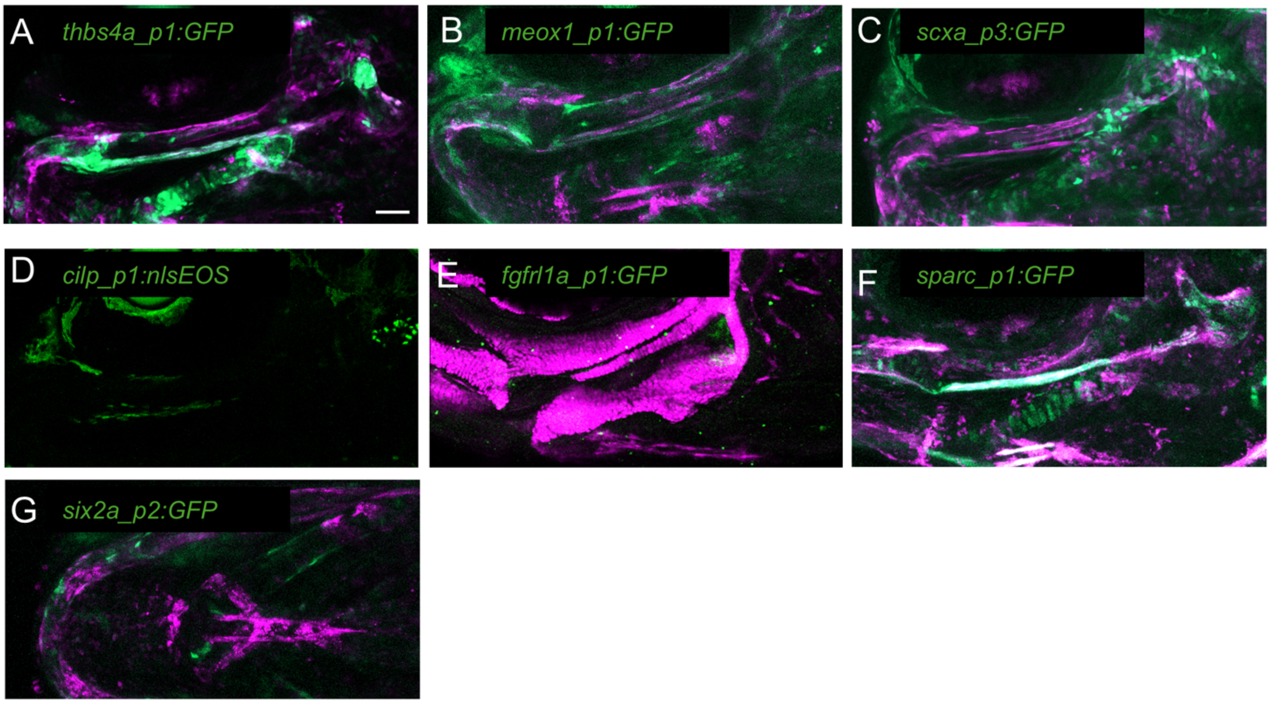

Fig. S5. Independent transgenic founder expression patterns

(A-G) Examples of independent alleles for the indicated transgenes related to Figure 6. Confocal projections of the face in lateral view show enhancer transgene expression (green) relative to tendons and ligaments (*scxa:mCherry*, magenta, A,B,C,F,G) or cartilage (*sox10:DsRed*, magenta, E). Scale bars = 50µm

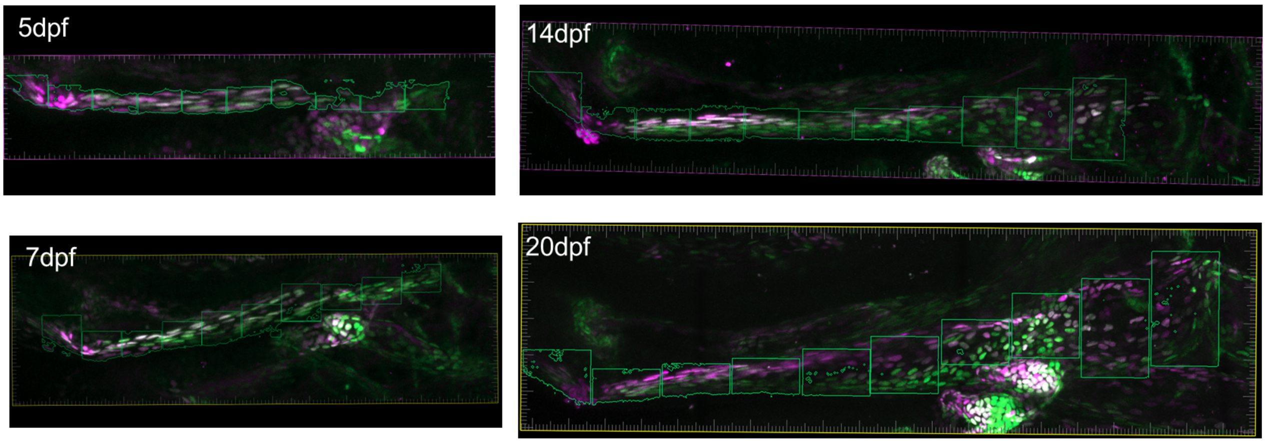

Fig. S6. Segmentation for quantitation of anterior-posterior mc-op ligament growth

Representative examples of the anterior-posterior segmentation used to quantitate the green/red fluorescence ratio. Green boxes correspond to segments of Fig. 7.

Development • Supplementary information

Table S1. Marker genes for connective tissue clusters

Available for download at
https://journals.biologists.com/dev/article-lookup/doi/10.1242/dev.205045#supplementary-data

Table S2. Genomic coordinates of tested DARs

Available for download at
https://journals.biologists.com/dev/article-lookup/doi/10.1242/dev.205045#supplementary-data

Development • Supplementary information
